# Sunday Entertainments

**Published:** 1899-11-18

**Authors:** 


### The Hospital

A Journal of

The Medical Sciences and Hospital Administration.

Vol. XXVII.—No. 686. Edited by Sir Henry Burdett, K.C.B., and Solomon C. Smith, M.D., M.R.C.P. [November 18, 1899.

### SUNDAY ENTERTAINMENTS.

If the spirit of Voltaire were free to exercise itself about sublunary things, it could scarcely find a more congenial subject for meditation than the annually recurring proceedings of the Licensing Committee of the London County Council, engaged in the work of treating its constituents as if they were children, incapable of deciding for themselves as to the entertainments which should be patronised and the entertainments which should be avoided. There is a case on record of a cantankerous old lady who was stricken down by paralysis, and who, during the last three months of her life, retained only sufficient power of articulation to reiterate without ceasing, "I won't—you shan't." The physical condition to which she was reduced is not an unfair representation of the mental condition of certain classes of the community, classes very much represented in the Council, in whose eyes it is improper for ginger to be hot i' the mouth, and who would fain, like their prototypes immortalised in Hudibras, exclude all other persons from enjoyments to which they themselves have no inclination. Very few things have given more innocent pleasure, in the course of the last year or two, than the many kinds of musical performance which have been organised for Sundays. In this organisation the London County Council has taken a conspicuous part, and its bands perform on Sunday afternoon in various open spaces within its jurisdiction. These performances are attended by large numbers of well-behaved and neatly-dressed members of the lower middle and working-classes, husband and wife bringing their children, and meeting their friends and acquaintances, and seats being provided by the Council at the modest charge of one halfpenny. "Sunday labour" is, of course, involved; the bandsmen, who are mostly in uniform, being paid, and a certain amount of attendance being necessary, both for the collection of the chair-rent and as a precaution against the possibility of disorder. It is not too much to say that the tendency of these performances is to keep men out of public-houses, to strengthen domestic ties, to teach good manners and mutual forbearance to children, and to afford a wholesome and elevating recreation which would not otherwise be within the reach of the audiences. All this, however, depends manifestly upon weather; and it was a natural consequence of the action of the Council in providing instrumental music in the open that various societies and persons should seek to make similar provision under cover, so as not only to meet the contingency of wet days, but also to facilitate the addition of vocal music to the programme. The National Sunday League and other bodies were soon in the field, and various concerts under cover were established by their enterprise.

Mr. Lecky, in his admirable account of persecution, describes how, in every country, its prevalence and its severity have been in precise proportion to the power wielded by the clergy, to whatever denomination they might belong; and one of the first consequences of the Sunday concerts was to stir up the ministers of various denominations in angry protest against any other Sunday occupation than that of listening to preaching. It seems to be provided by some statute or some bye-law that public concert-rooms, if used on Sunday, must not be so used as to yield a profit to their proprietors; and on this wonderful basis not only has the law been set in motion, but also the Licensing Committee of the County Council has been asked to refuse licences to establishments in which any such Sunday profit has accrued. It is quite evident that such a course would be prohibitory of the concerts. One person more or less in the audience would suffice to convert a loss of threepence into a profit of the same amount; and no proprietor could reasonably be expected to take the trouble of sailing sufficiently close to the wind to render profit impossible. But the Licensing Committee, in dealing with the Crystal Palace Company, was not satisfied with a general statement that the loss on the Sunday concerts for the season was £386, or that a hoped-for surplus, which was to have been handed to the Prince of Wales's Hospital Fund, has not accrued; and they deferred the consideration of the question for a week, in order to be informed of the amount paid in royalties by the refreshment contractor. In other cases that came before them, a like spirit of puerile illiberality and a like desire to dictate to their neighbours have been equally conspicuous. Voltaire described how the country priests of his day in France prohibited on holy days, under stress of multiplied curses, even the most necessary work, such as that of saving a corn crop when bad weather seemed to be impending, but had no word of reproach for the drunkenness which was the only recreation left to the peasantry. Our municipal wiseacres would in like manner reduce a wet Sunday within their boundary to the level of a Scottish one, on which a closed door and a whisky bottle furnish the only available resources against the tedium of enforced idleness. We hope the Council as a whole will be wiser than its committee, and will recognise the great truth that freedom, and a sense of responsibility for its employment, furnish the first and most essential conditions of moral improvement and of social elevation.

